# Clinical and Instrumental Characterization of Patients With Late-Onset Epilepsy

**DOI:** 10.3389/fneur.2022.851897

**Published:** 2022-02-25

**Authors:** Jacopo C. DiFrancesco, Angelo Labate, Michele Romoli, Elena Chipi, Nicola Salvadori, Carlo Andrea Galimberti, Daniela Perani, Carlo Ferrarese, Cinzia Costa

**Affiliations:** ^1^Department of Neurology, ASST S. Gerardo Hospital, School of Medicine and Surgery and Milan Center for Neuroscience, University of Milano - Bicocca, Monza, Italy; ^2^Neurophysiopathology Unit, Department of Biomedical and Dental Sciences, Morphological and Functional Images (BIOMORF), University of Messina, Messina, Italy; ^3^Section of Neurology, S. Maria della Misericordia Hospital, Department of Medicine and Surgery, University of Perugia, Perugia, Italy; ^4^Epilepsy Center, IRCCS Mondino Foundation, Pavia, Italy; ^5^Nuclear Medicine Unit and Division of Neuroscience, San Raffaele Hospital, Vita-Salute San Raffaele University, Milan, Italy

**Keywords:** late-onset epilepsy, Alzheimer's disease, brain MRI, electroencephalography, cerebrospinal fluid, neuropsychology, positron emission tomography, computed tomography

## Abstract

Epilepsy is classically considered a childhood disease. However, it represents the third most frequent neurological condition in the elderly, following stroke, and dementia. With the progressive aging of the general population, the number of patients with Late-Onset Epilepsy (LOE) is constantly growing, with important economic and social consequences, in particular for the more developed countries where the percentage of elderly people is higher. The most common causes of LOE are structural, mainly secondary to cerebrovascular or infectious diseases, brain tumors, trauma, and metabolic or toxic conditions. Moreover, there is a growing body of evidence linking LOE with neurodegenerative diseases, particularly Alzheimer's disease (AD). However, despite a thorough characterization, the causes of LOE remain unknown in a considerable portion of patients, thus termed as Late-Onset Epilepsy of Unknown origin (LOEU). In order to identify the possible causes of the disease, with an important impact in terms of treatment and prognosis, LOE patients should always undergo an exhaustive phenotypic characterization. In this work, we provide a detailed review of the main clinical and instrumental techniques for the adequate characterization of LOE patients in the clinical practice. This work aims to provide an easy and effective tool that supports routine activity of the clinicians facing LOE.

## Introduction

Epilepsy is classically considered an infantile-pediatric disease. However, the onset of the disease in the adulthood, after the age of 50, is common. Following cerebrovascular diseases and dementia, epilepsy is in fact the most frequent neurological condition affecting elderly subjects (1). Numerous studies showed that, since the adulthood, there is a progressive and constant increase in both the incidence and prevalence of epileptic seizures and epilepsy in the general population (2). This is independent of the socio-cultural and economic context examined, affecting both developed and low-income countries (3). In particular, in the richer communities (i.e., Europe and USA), where the majority of the population consists of old people, epilepsy with onset in the elderly has significant economic and social repercussions (4). Furthermore, with the global improvement of living conditions and access to care, a progressive increase in the aging population is expected in the future also for developing countries, with a consequent increase of the number of patients with Late-Onset Epilepsy (LOE).

In most cases, LOE is characterized by focal seizures, secondary to brain damage, particularly cerebrovascular and infectious diseases, brain tumors, metabolic, and toxic conditions (3). More recent evidence reports that LOE can also be associated with neurodegenerative conditions, particularly Alzheimer's disease (AD). While it is well-known that AD patients have an increased risk of epilepsy during the course of the disease (5), recent data show that LOE may represent the first clinical manifestation of AD. In these subjects, the onset of epilepsy may precede the development of cognitive symptoms by years, while the pathophysiological markers of AD are already positive (6–13).

In the clinical practice, patients with LOE are subjected to first-level clinical and instrumental investigations that allow the identification of the causes of the disease in the majority of cases. In particular, medical history, neuroradiological examinations, electroencephalography (EEG) and hematochemical tests allow an adequate characterization of the phenotype in most of the patients. However, in a subgroup, these first-level examinations are not sufficient, and more in-depth investigations are necessary to improve the diagnostic accuracy. The application of this second-level approach should be rationally definite case-by-case, based on the specific orientation for the patient. Moreover, despite a careful phenotypic characterization, the causes of the disease are not identified in a variable portion of patients (8). To date, detailed epidemiological data regarding LOE are missing. Population studies report that the incidence of new cases with no identifiable etiology ranges from 25 to 50% (14–16). These subjects are therefore defined as Late-Onset Epilepsy of Unknown origin (LOEU) (17, 18).

In the common clinical practice, several investigations are used for the characterization of LOE patients. Their exhaustive characterization is important in order to adapt treatments and diagnostic procedures, avoiding unnecessary if not harmful therapies and tests, and to assess a correct prognosis. In this work, we provide a detailed revision of the principal clinical and instrumental techniques for the adequate characterization of LOE in the clinical practice. The purpose of this work is to offer an easy and effective tool supporting the routine activity of the clinicians facing LOE.

## First Level Examinations

In the routine clinical activity, patients with LOE are subjected to several first level investigations with the aim to identify the most common causes of LOE (Table 1). In most cases, these examinations allow the correct clinical-diagnostic classification of patients. Accordingly, first level analyzes should be performed in all patients with a first diagnosis of LOE.

**Table 1:** Characterization of patients with late-onset epilepsy.

**Analysis**	**Specific assessment**	**Objective**	**Focus on**
**First line examinations**			
Medical history	Descriptions of the seizures by the patient and witnesses	Characterization of the epileptic phenotype, identify the epileptogenic focus/i	Onset/lateralization of symptoms and subsequent spread, aura, alteration of consciousness, other specific symptoms useful to identify the epileptogenic focus
	Collection of information from the patient and/or relatives	Identification of risk factors for epilepsy (past or present)	Alcohol abuse (acute or chronic), acute withdrawal of chronic pharmacological treatments (e.g., benzodiazepines), drugs of abuse, neuroleptics, family history of epilepsy, previous epilepsy (infantile/pediatric), electroshock, major head trauma (with coma)
Hematochemical tests	Electrolytes, liver and kidney function, hormone levels (especially thyroid and parathyroid), neoplastic markers	Identification of metabolic alterations responsible for epilepsy	Electrolyte imbalance, major hormonal changes, kidney or liver failure, hyperammonaemia, increase of neoplastic markers
Cardiovascular and pulmonary	Heart rhythm analysis (ECG, ECG-Holter, tilting test)	Differential diagnosis with alterations of the heart rhythm (mainly lipothymia, syncope)	Major changes in heart rhythm (atrial fibrillation, prolonged pauses)
	Nocturnal polysomnography with oximetry	Sleep apnea	Obstructive sleep apnea syndrome (OSAS)
Neuroradiology	Brain CT scan	Study of the parenchyma	Identification of brain lesions responsible for epilepsy
	Brain MRI (T1 before/after contrast enhancement, T2/FLAIR, DWI sequences)	Past or acute cerebral lesions responsible for seizures	Brain tumors (benign or malignant), cerebrovascular disease (ischemic or hemorrhagic), head trauma, brain infection
	Brain MRI (T1-inversion recovery)	Cortical epileptogenic malformations	Mainly hippocampal sclerosis and cortical migration defects
	Brain MRI (T2-star/GRE/SWI)	Small vessel diseases	Cerebral microbleeds and cortical superficial siderosis, indicative of Cerebral Amyloid Angiopathy (CAA)
	Brain MRI (DWI/ADC)	Active cerebral lesions	Acute ischemic stroke, Creutzfeldt-Jakob disease (CJD)
	Brain CT/MRI angiography	Study of cerebral vessels	Stenosis of the cerebral vessels (DD with transient ischemic attack)
EEG	Standard EEG recording (S-EEG) and 24-h Ambulatory EEG (A-EEG)	Epileptiform and lesional activity	Background activity, effect of sensory stimulations (mainly HP and SLI), effect of sleep deprivation and activity during sleep
	Video-EEG	Subclinical seizures (gold-standard for PNES)	Correlation between epileptic activity and clinical expression of seizures
**Second line examinations**			
CSF	Cell and protein count, glycorrachia, batterioscopic and viral examinations	Infection of the CNS	Viral and bacterial meningoencephalitis
	Analysis of the principal neurodegenerative biomarkers	Neurodegenerative disease (MCI, AD) and prion disease (CJD)	Reduction of Amyloid-β 1-42 level, increase of tau and phospho-tau (P-tau), dosage of 14-3-3 protein, RT-QuIC
	Dosage of antibodies against neuronal surface targets	Antibody-mediated autoimmune encephalitis	Specific autoantibodies against neuronal surface targets (most frequent LGI1, NMDAR, and GABA_B_ R)
Neuropsychology	MiniMental State Examination (MMSE), HIV-Dementia Scale (HDS), Digit span, Rey Auditory Verbal Learning Test (RAVLT), Category and Letter fluency, Frontal Assessment Battery (FAB), Copy of Drawings with/without Landmarks (CD/CDL) or Rey-Osterrieth Complex Figure (ROCF), Symbol Digit Modalities Test (SDMT), Raven's Progressive Standard Matrices'47 (PM'47)	Neurodegenerative disease (mainly MCI and AD), also at a presymptomatic stage	Deficit in one or more of the following areas: global cognition, verbal memory, language, attention/executive functions, logical reasoning, visuo-spatial abilities
Quantitative EEG analysis	Connection between regions (segregation) and length of pathways (integration)	Study of brain cortical connectivity	Modeling of brain regions and their connections to define the mathematical representation of brain connectivity (“small world”-SW)
Nuclear medicine	Brain ^18^F-FDG-PET	Measurement of brain glucose metabolism	Useful for the identification of the epileptogenic focus; diffuse or localized hypocaptation of the tracer (in the diagnostic investigation of AD and other dementias)
	Amyloid-β (Aβ) PET	Measurement of cortical Aβ deposition	Increased Aβ deposition (diffused or localized)
	Hybrid PET/MRI	Simultaneous measurement of brain tissue and metabolic changes	Useful for the identification of the epileptogenic focus, cortical malformations, dementia

### Medical History

The first step for the correct characterization of the patient with LOE is the collection of information about the characteristics of the seizures, both directly from the patient and from relatives/witnesses. In particular, it is important an accurate collection of the objective and subjective manifestations of the episodes, when possible, with the support of video clips of the clinical manifestations, in order to facilitate the correct interpretation of the episodes. It is also important the identification of any risk factors for the seizures, considering those mainly typical of the adulthood/old age. In particular, clinicians should exclude the effect of alcohol abuse or withdrawal, and the presence of drug therapies that can facilitate the onset of epileptic seizures, especially benzodiazepines and neuroleptics, but also some antibiotics and other commonly used medications. Moreover, by thoroughly investigating the remote medical history of the patient, also including childhood period, it is important to exclude a past history of epilepsy.

### Hematochemical Tests

Commonly in the elderly subjects, metabolic alterations, particularly electrolyte imbalance (19, 20), can manifest with seizures, also at high frequency or even with *status epilepticus* (21). These include the reduced level of the principal serum electrolytes: sodium (<115 mg/dl), calcium (<5.0 mg/dl), and magnesium (<0.8 mg/dl). Other common conditions are represented by the alteration of metabolic homeostasis: low (<36 mg/dl) or high (>450 mg/dl) serum glucose, reduced urea nitrogen concentration (<100 mg/dl) and high creatinine level (>10.0 mg/dl). Since these alterations are easily treatable, they should be promptly identified and adequately corrected.

Other conditions predisposing for the onset of seizure, also typical of the adult age, are represented by alcohol withdrawal and/or intoxication (22, 23) and the intake of illicit drugs, typically cocaine (24). When clinically suspected, the measurement of plasma alcohol levels and the search for drugs metabolites in urine or blood are important data in the diagnostic process.

### Cardiovascular and Pulmonary

In the adult-elderly age there are various conditions linked with episodic loss of consciousness. Aside seizures, other potentially life threatening diseases should be excluded (25). The main causes of non-neurological loss of consciousness, sometimes difficult to discriminate from epileptic seizures, are secondary to the alteration of the cardiovascular system. Major changes in the heart rhythm, such as atrial fibrillation and prolonged pauses, can result in lipothymia and syncope (26, 27). It is therefore essential to analyze the heart rhythm with techniques such as ECG, ECG-Holter, and tilting test.

Furthermore, chronic alterations of respiratory mechanics, in particular obstructive sleep apnea syndrome (OSAS), can predispose to the development of epileptic seizures. In fact, the chronic hypooxygenation of the brain favor the development of epilepsy (28). In subjects with OSAS, the analysis of nocturnal polysomnography with oximetry is important in order to verify the extent of sleep apnea.

### Neuroradiology

Neuroradiological investigations, in particular brain Magnetic Resonance Imaging (MRI), are essential to identify any structural damage responsible for the onset of seizures (29). Although, Computed Tomography (CT) remains the neuroimaging method of choice in many countries, constant advances in MRI technology led to dramatic advances in the obtaining high quality information about the living brain, which had previously only been possible by postmortem examination (30).

Evidence-based guidelines from the American Academy of Neurology recommend immediate non-contrast CT in emergency patients presenting with a first seizure regardless age at onset (31). This is still particularly important in elderly subjects to guide appropriate acute management, especially in situations such as a hemorrhage, stroke, calcified lesions or tumors.

Even if CT scan remains of value in the absence of MRI and should be considered the neuroimaging procedure of choice under these circumstances, since mid 1980s, brain MRI is largely applied for a better characterization of the epileptic phenotype (32). In the last few years, the scientific international community proposed a standard typical protocol for epilepsy-specific structural imaging based on a balance between diagnostic accuracy and clinical feasibility (33). The main advice is that neuroimaging workup of patients with epilepsy requires a minimum set of MRI basic sequences (available on most MR scanners), regardless of the clinical setting and country. Very recently, the Neuroimaging Task Force, has emphasized the meaning of high spatial resolution and image contrast with complete brain coverage to optimally assess brain anatomy and suggested the use of a three-dimensional acquisition called the harmonized neuroimaging of epilepsy structural sequences (HARNESS-MRI protocol) (34, 35). T1 before/after contrast enhancement, T2/FLAIR and DWI sequences are useful for the identification of past or acute cerebral lesions responsible for seizures; T1-inversion recovery sequence for cortical epileptogenic malformations such as cortical dysplasia and hippocampal sclerosis. Moreover, three-dimensional (3D) sequences with isotropic voxels of 1 mm or less dramatically reduce partial volume effects, a phenomenon resulting from the presence of multiple tissue types within a given voxel. Remarkably, partial volume is unfavorable when looking for subtle cortical dysplasia, as it mimics tissue blurring, a cardinal feature of these lesions. Even the application of T2-star/GRE/SWI sequences are important for the detection of small vessel diseases, both ischemic and hemorrhagic, as Cerebral Amyloid Angiopathy (CAA) and CAA-related inflammation (36, 37). In the differential diagnosis between seizures and transient ischemic attack (TIA), it is important to carry out a study of the cerebral vessels by means of CT angiography or magnetic resonance angiography.

Furthermore, imaging and post-processing imaging helps to identify early structural alterations preceding the development of drug-resistance in mild mesial temporal lobe epilepsy with LOE and to better understand pathophysiology in such syndrome (38–41). However, it should be considered that the possibility of identifying some lesions (e.g., focal cortical dysplasia) in patients with LOE is lower compared to younger subjects (8, 42, 43).

Very recently, hybrid PET/MRI systems have the potential to combine the superior soft-tissue contrast of MRI and the metabolic features of FDG-PET in a single exam (44). This may be an ideal imaging tool for detection of the epileptic zone because it can evaluate both anatomical and functional information simultaneously. This new procedure can also be very useful to study subjects with LOE and cognitive disorders such as dementia (45).

### Electroencephalography (EEG)

Detection of interictal EEG epileptiform discharges (IEDs) might be crucial to support a correct diagnosis in elderly, which are more likely than younger adults to present paroxysmal phenomena that masquerade as epileptic seizures. A standard EEG recording (S-EEG) is commonly the first diagnostic step in the suspect of epileptic seizures of recent onset. Nevertheless, some data in the literature indicate a reduced S-EEG sensitivity, with a lower rate of IEDs in older epilepsy patients (46–48). Moreover, both seizure etiology and comorbid medical condition (49–51), as well as several drugs, may affect IED incidence.

Among activation procedures, an adequate duration of sleep EEG recordings seems to increase the yield of IEDs in both young and elderly epilepsy patients (49). An early assessment by 24-h home-recorded Ambulatory EEG (A-EEG) in drug-free adults with recent onset focal LOEU, showed a higher propensity in elderly patients than in younger age for spiking mainly or exclusively during deep stages of NREM sleep (48). This condition appears difficult to achieve in elderly subjects by day-time laboratory EEG recordings during spontaneous or induced sleep (47). In addition, A-EEG significantly increases the probability to detect ictal epileptic discharges (52), thus it would be opportune in elderly subjects also in order to confirm a diagnosis of epileptic seizures and to verify their possible recurrence.

Although differences in seizure features between elderly and younger adult are matter of debate (53, 54), semiology and recurrence of epileptic seizures might be more difficult to ascertain in older patients. Both physiological and psychogenic non-epileptic seizures, alone or epilepsy-associated, are probably a frequent problem in elderly. Several studies (55–59), highlighted feasibility and usefulness of Video-EEG monitoring in diagnosis and differential diagnosis of seizure disorders in elderly; nevertheless, this subpopulation appears to be underrepresented among patients referred to epilepsy centers for this type of investigation.

## Second Level Examinations

**Figure 1:**
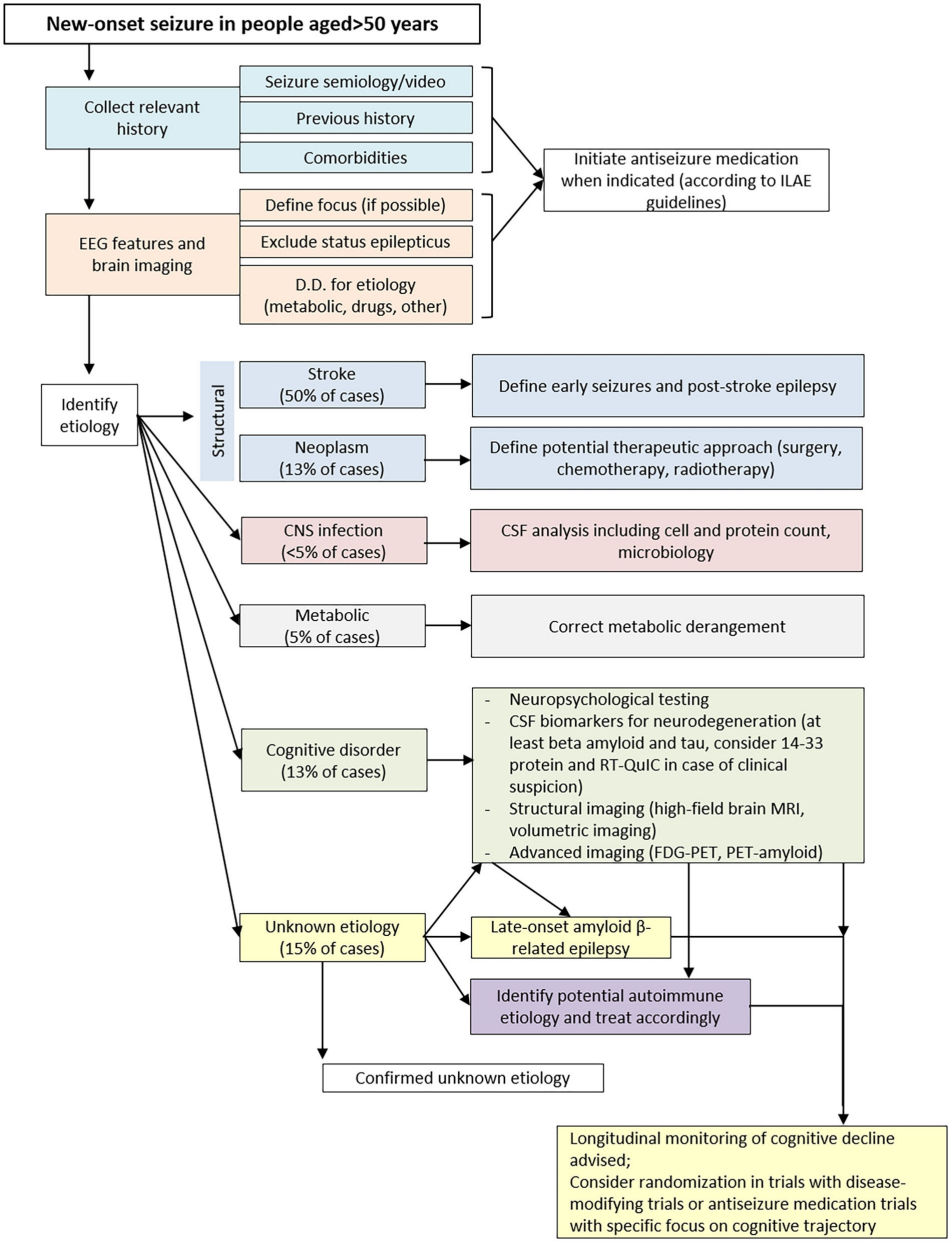
Flow-chart for the characterization of patients with LOE.

Despite the application of the first level methods discussed above, the causes leading to late-onset seizures are not identified in a considerable percentage of subjects. In this case, it is important that these patients undergo second level investigations (Figure 1). Contrary to the first level methods, to which all patients should be subjected, the most in-depth analyses should be selected case by case, according to the diagnostic orientation of the clinicians.

### Cerebrospinal Fluid (CSF)

The analysis of cerebrospinal fluid (CSF) can have numerous applications, based on the clinical-instrumental orientation specific to the case studied. The most common application of CSF analysis relates to the suspicion of Central Nervous System (CNS) infection, such as viral or bacterial meningoencephalitis. Into the correct clinical context, the analyses focus on cell and protein count, glycorrachia, bacterioscopic, and viral examinations.

Another important application in the diagnostic process of LOE relates to the evaluation of biomarkers of amyloidosis, tauopathy and neurodegeneration. In the suspicion of a neurodegenerative disease, in particular MCI due to AD, it is important to measure the concentration of the principal biomarkers of amyloidopathy (level of amyloid-β 1-42 protein) and axonal damage (TAU and P-TAU). Moreover, if prion encephalopathy (CJD) is suspected, the assay of the protein 14-3-3 and RT-QuIC analysis in the CSF is a valid support to the diagnosis.

The analysis of CSF is also fundamental for Antibody-Mediated Autoimmune Encephalitis (AMAE) (60). Classically in these encephalopathies, patients present with rapidly progressive epilepsy, poorly responsive to antiseizure drugs (ASDs). The disease is sustained by specific autoantibodies directed against neuronal surface targets (61). The most common forms are due to antibodies against LGI1, NMDAR and GABA_B_ R (62). It is important to recognize AMAE and treat them promptly with immunosuppressive therapy to improve patient prognosis and outcome.

### Neuropsychology

Over the last 20 years, most of neuropsychological studies were carried out in adult patients with chronic epilepsy, reporting consistent rates of cognitive impairment (63–67). The most frequent findings were memory impairment, mental slowing and attentional deficits (64, 68).

More recent studies found that cognitive deficits are very common in patients with LOE, even at time of diagnosis (69–71). The assessment of the neuropsychological profile plays a significant role in the diagnostic process of LOE, mainly in the suspicion that the onset of seizures could be the first manifestation of a neurodegenerative disease. This applies is particular to those with LOEU that, in some cases, may represent a feature of an underlying AD condition (7–9, 11, 12, 17, 72, 73).

Up to now, in clinical practice, there is no agreement regarding neuropsychological assessment, with test batteries and timing being the most crucial issues. Most epilepsy centers use different batteries, resulting in substantial heterogeneity regarding test selection and outcomes (12, 73). Measures frequently used include test of global functioning, verbal episodic memory, visuospatial memory, attention/working memory and executive functions, word fluency, visuospatial functions, and constructional praxis, and logical-perceptual abilities (7–9, 11, 17, 63–67, 74, 75).

Standardizing neuropsychological assessments for detecting cognitive impairment in LOE is a priority, since the use of different measures necessary leads to different outcomes (70, 76). The only screening tool specifically designed to detect and monitor cognitive aspects in patients with epilepsy is EpiTrack (77), a brief task including tests of attention and executive functions (i.e., response inhibition, visuomotor speed, mental flexibility, visuomotor planning, verbal fluency, and working memory). EpiTrack was originally built to evaluate long-term cognitive side effects of ASDs, and its reliability in tracking global cognitive function is still largely unexplored. To this extent, although EpiTrack represents a promising tool to monitor ASDs side effects, its use is limited as it leaves other domains unexplored (78). Such point can be critical, since deficits in LOE impact on multiple cognitive domains rather than being focused on only one (7, 9, 63, 64, 66, 73, 74, 79). A neuropsychological assessment limited to screening and/or evaluation of executive functions only can lead to an underestimation of cognitive functioning in other domains (69). Therefore, a standardized and comprehensive evaluation assessing the wide spectrum of cognitive domains (i.e., global cognition, memory, attention/executive functions, language, logical reasoning, and visuospatial skills) is needed during the diagnostic work-up of LOE patients (8).

In Table 1 we report a proposal of extensive neuropsychological battery including the most used cognitive tests available in literature, potentially applicable in all centers. To the most used tests we would suggest to add the HIV-Dementia Scale (HDS), a screening tool able to detect cognitive deficits with prevalent subcortical pattern, being complementary to MMSE in clinical practice (80). Moreover, a certain degree of discrepancy may occur in some cases between neuropsychological scores and the clinical judgment on cognitive status. To this purpose, we propose to add the Clinical Dementia Rating (CDR) scale, in order to stage clinical status.

At last, some methodological issues should be carefully taken into account in the administration of test batteries. In general, since polytherapy with ASDs may influence scores on different domains, cognitive status should be always assessed at the time of diagnosis (81). This allows to identify those who manifest cognitive impairment before starting ASDs, providing a baseline from which measure the cognitive change of the disease over time and/or the effects of subsequent treatment (66, 74, 76, 82).

Longitudinal examinations of cognitive profile are recommended to monitor clinical trajectories over time and the outcomes of ASDs. Those assessments are best be performed following an adequate interval from the last seizure or when the seizure management is stable, or before the start of ASDs. We advise against repeating neuropsychological assessment within 6–9 months, due to practice effect and potential inconsistent estimates in cognitive trajectory. This is particularly true for measures of attention, memory, speed of information processing, and higher-level executive functions (83). Serial cognitive testing with long follow-up periods, especially in patients with evidence of abnormally low CSF Aβ levels, can be useful for the stratification of risk to develop cognitive decline or dementia in this population (12, 73).

### Quantitative EEG Analysis for the Study of Brain Connectivity

A further step in EEG application in LOE is related to advanced analysis of cortical connectivity (7). EEG network analysis consists in the modeling of brain regions and their connections into a system characterized by two main features: type of connection between regions, defined as segregation, and length of pathways, defined as integration. The more the brain regions are intertwined, the more they segregate one another. At the same time, the connections of each brain region are far from being univocal, and therefore the model takes into account how the anatomical pathways between brain regions can participate in generating EEG activity. Balancing segregation and integration EEG advanced analysis can provide a mathematical representation of brain connectivity, defined as “small world” (SW) (84–86). Since cognitive decline is associated with the loss of brain connectivity, SW analysis and EEG connectivity has been investigated as a potential tool to identify people at higher risk of dementia. EEG-related neural networks analysis with SW approach demonstrated ability to distinguish Mild Cognitive Impairment (MCI) at risk of progression to AD from the general population (84).

Recently, EEG connectivity has been investigated also in LOE. By now, only few studies investigated the role of EEG connectivity analysis in LOE patients, with particular regard to LOEU (7, 13). Preliminary evidence from small sample studies highlight that cortico-cortical network is already altered at the time of epilepsy diagnosis among those people having the highest risk of dementia. In particular, SW features of people evolving to dementia over a 5-year time were already substantially different from those preserving normal cognitive function over that time (7, 13). SW reduction in the theta and delta frequency bands could be considered a potential marker of subtle cortico-cortical disconnection, potentially identifying people with LOE at the highest risk of conversion to dementia in the years following epilepsy diagnosis (7, 13). Since SW is affordable and available in any setting, SW analysis could be proposed as potential tool to provide a stratification of the risk of dementia, potentially predicting cognitive decline among patients with LOE.

### Nuclear Medicine

Positron Emission Tomography (PET) is useful for clinical studies in epilepsy, and specific ligands probe the pathophysiology of brain dysfunction, neurochemical and receptor abnormalities. PET with 18F-FDG has been used for over 40 years to help identifying regions of focal cerebral hypometabolism, with an output of about 60% for temporal lobe epilepsy and 30% in extratemporal lobe epilepsies. FDG-PET, measuring glucose metabolism in the brain, is commonly used in the pre-surgical process for drug-resistant focal epilepsies. Dynamic PET acquisition with a quantitative assessment, rather than static PET imaging, has been suggested to increase sensitivity in identifying areas of hypometabolism when conventional FDG-PET and MRI are unremarkable (87).

Within the second level examinations in the diagnostic process of LOE, i.e., the epilepsies with onset after age 50, FDG-PET is rarely assessed. However, we should consider that brain FDG-PET is widely used to identify specific brain areas of glucose hypometabolism in neurodegenerative processes, often associated with an epilepsy syndrome (8). Together with clinical features, including neuropsychological profile, and laboratory data, in particular CSF biomarkers of amyloidosis, tauopathy and neurodegeneration, the presence of cerebral hypometabolism leads to the diagnosis of specific dementia subtypes (88). Of note, Aβ PET can mark the presence of Aβ protein in the brain parenchyma, thus supporting the diagnosis of Alzheimer's disease in patients with LOE.

Besides clinical applications, PET with adequate ligands is a highly specialized technique actively used to clarify the pathophysiology of epilepsy and possibly identify novel treatment targets. The most active lines of research are particularly focused on the relationship between inflammation, microglial activation, and epileptogenesis. In fact, brain injury and proconvulsant events can activate microglia and astrocytes to release a number of proinflammatory mediators leading to a cascade of neuroinflammatory processes in the brain. There is evidence for the implication of glial cells in a number of molecular mechanisms of seizure precipitation and recurrence: alterations in the phenotype and function of activated microglia and astrocytes, alterations of glutamine/glutamate cycle and glutamate receptor expression, release of neuromodulatory and neuroinflammatory molecules (89). PET with translocator protein (TSPO) showed an association with microglial activation, astrogliosis and cell death. So far, imaging of neuroinflammation in the context of epilepsy has been performed in limited cases, supporting the view that activated microglia, reactive astrocytes, and inflammatory intermediates may contribute to hyperexcitability in seizure foci. Increased uptake of [11C]-(R)-PK11195, or [11C]PBR28, both TSPO tracers, was observed in regions of epileptic foci and together with focal cortical dysplasia as determined by high resolution MRI, electroencephalography recording and intraictal and interictal [18F]FDG-PET contributes in demonstrating the integrated pathological role (90, 91). These findings confirm results from invasive studies revealing a specific and persistent increase in the numerical density of activated microglia within dysplastic regions of patients with epilepsy, and support the view that the inflammatory response and proinflammatory molecules contribute to epileptogenicity of focal cortical dysplasia (92). Clinical studies with more recently developed radioligands such as [11C]- PBR28, found increased TSPO ipsilateral to seizure foci in temporal lobe epilepsy, even if further studies are needed to evaluate the clinical role of this ligand (93).

Synaptic vesicle glycoprotein 2A (SV2A) binding is reduced in brain specimen obtained by epilepsy surgery. *In-vivo* binding of the SV2A PET tracer [11C]UCB-J was reduced in hippocampal sclerosis, with the reduction being greater than what accounted by hippocampal atrophy and reduction in FDG uptake, suggesting a further molecular process (94). A novel PET tracer, derived from 4-[2-(phenylsulfonylamino) ethylthio]-2,6-difluoro-phenoxyacetamide labeled with 11C ([11C]K-2), was recently developed that specifically binds to AMPA receptors *in vivo*. Increased binding was found in the cortex of patients with mesial temporal lobe epilepsy, which correlated with the AMPA receptor protein distribution in resection specimens (95). The use of PET tracer that can visualize and quantify AMPA receptors *in vivo* is of potential great interest and importance in the investigation of the pathophysiology of the epilepsies and seizures.

## Discussion

In this work, we reviewed the main evidence available for a correct characterization of LOE, with the aim of providing clinicians with a useful tool for the management of first diagnosed patients. The diagnostic process of these subjects should be adapted case-by-case, based on the specific characteristics of the single individual. Due to the progressive aging of the population worldwide, particularly in more developed countries, there is an urgent need for a better understanding of the pathogenetic mechanisms underlying the development of epilepsy in adulthood. This is also useful for adequately planning the resources to be allocated by the NHS to these patients who will become more numerous in the near future.

While the causes of the epilepsies with infantile and pediatric onset are often genetically determined, in the adult population they are mostly secondary to structural alterations (3). However, non-structural causes may also be responsible for the late onset disease, as infection, but also autoimmune and neurodegenerative diseases. According to this, several techniques have been developed for a better characterization of patients with disease onset in adulthood that are here reviewed.

In the clinical activity, some diagnostic investigations are generally carried out routinely in all patients (first level), while for some subjects additional investigations are also needed (second level). Among the first investigations, in the recent years brain MRI considerably improved the diagnostic definition of patients. The application of uniform study protocols allowed the standardization and dissemination of advanced analysis methods to define the structural causes of the disease.

The EEG remains a fundamental test in the diagnostic process of epilepsy, although in adult subjects has a lower ability to identify IED compared to the pediatric population. Even if not routinely applied, more in-depth methods such as A-EEG and V-EEG are more effective in the diagnostic confirmation of epileptic activity. Recent scientific evidence also demonstrates that the study of the electrical signal can provide important data regarding segregation and integration of the brain activity, resulting in the graphical representation of brain connectivity. Thus, a simple and non-invasive examination such as EEG, easily repeatable in the follow-up of patients, can provide useful information about the evolution over time of brain electrical activity, thus furnishing a potential biomarker to monitor the conversion to dementia.

When routine investigations fail to determine the causes of LOE, further analysis is advisable. At this stage, one fundamental assessment is certainly the analysis of CSF. Although invasive and contraindicated in some cases (e.g., oral anticoagulant therapy), this investigation allows to identify several clinical conditions also very different from each other. The first application of CSF analysis is for the infectious of CNS (bacterial or viral meningoencephalitis) which can present with epileptic seizures even at an early phase of the disease (96, 97).

Moreover, the analysis of pathophysiological markers of amyloidosis, tauopathy and neurodegeneration can identify the predisposition to a subsequent evolution toward a condition as MCI due to AD. Although further confirmation is needed in larger case series, recent scientific evidence shows that LOE patients with an altered CSF profile, in particular low CSF Aβ level, have a greater chance of evolving to dementia in the years following the onset of epilepsy (9–13). CSF analysis is also important for those patients presenting with rapidly progressive epilepsy, associated with a severe clinical picture. The assay of 14-3-3 protein and RT-QuIC can confirm the suspicion of CJD. Moreover, the identification of specific autoantibodies directed against neuronal surface targets supports the diagnosis of AMAE. While to date the prognosis of CJD still remains poor, it is very important to quickly diagnose AMAE, which generally can benefit from immunosuppressive treatment.

In order to evaluate all the possible causes of LOE, it is important to include a thorough neuropsychological evaluation. Considering all the interactions with drugs active on the CNS (frequently used in elderly subjects), this non-invasive and easily replicable investigation allows to identify even mild cognitive impairment, which often do not emerge at the routine neurological evaluation. In fact, recent evidence shows that LOE patients can present a mild cognitive impairment, characterized mostly by multidomain cognitive deficits (8). In particular, at the onset of seizures, significant cognitive impairment often does not emerge, which may be more evident at the follow-up, thus highlighting the importance of a periodic patient monitoring. In order to investigate all cognitive aspects, an exhaustive and thorough neuropsychological battery is necessary. However, to date there are still no specific guidelines regarding the tests to include in the assessment of LOE. In this review, we collected and discussed the main evidence available in literature for a comprehensive evaluation of patients.

In this scenario, the techniques of nuclear medicine also find application, both in the diagnostic process of LOE and for research purposes. Brain FDG PET is already routinely used for a better definition of the epileptogenic focus in epilepsy surgery. Moreover, data supporting a correlation between LOE and neurodegenerative diseases, in particular AD, allows to hypothesize that in the future this technique will be used more in the diagnostic process of these patients, together with hybrid PET/MRI. Other promising analysis techniques are in an active experimentation phase, with the aim of improving knowledge relating to the pathogenetic mechanisms of the disease; however, they are not yet currently in use in clinical practice.

## Conclusions

Overall, in the recent years the knowledge about LOE has significantly grown, with particular focus on the pathogenetic mechanisms of the disease. However, many of the innovative aspects reported in this review derive from current research, mostly based on small patient series. Only recently in fact emerged the higher predisposition of patients with LOE to develop neurodegenerative diseases, thus highlighting the importance of including in the characterization of patients also an in-depth evaluation of the neuropsychological profile, CSF biomarkers and the application of techniques of nuclear medicine. Overall, these aspects still need to be further confirmed and investigated.

It is important that all patients with a diagnosis of LOE undergo an adequate phenotypic characterization and periodic follow-up. However, it also must be considered that, despite an adequate and exhaustive characterization with the most innovative methods, a significant portion of these cases remains without an etiological diagnosis regarding the causes of the disease. For these reasons, it is important to broaden the knowledge in this innovative field of research, through targeted studies of large patient series. In the future, this will pave the way to establish selected treatment pathways, based on the specific causes of the disease.

## Author Contributions

JCD and CC contributed to conception and design of the review and wrote the first draft of the manuscript. AL, MR, EC, NS, CG, DP, and CF wrote sections of the manuscript. All authors contributed to manuscript revision, read, and approved the submitted version.

## Conflict of Interest

The authors declare that the research was conducted in the absence of any commercial or financial relationships that could be construed as a potential conflict of interest.

## Publisher's Note

All claims expressed in this article are solely those of the authors and do not necessarily represent those of their affiliated organizations, or those of the publisher, the editors and the reviewers. Any product that may be evaluated in this article, or claim that may be made by its manufacturer, is not guaranteed or endorsed by the publisher.
